# Who are the speech and language therapists who work with primary progressive aphasia in Brazil? An exploratory cross-sectional survey study

**DOI:** 10.1590/1980-5764-DN-2023-0016

**Published:** 2023-12-11

**Authors:** Amanda Gorziza da Silva, Eduardo Kunz Beux, Gabriela Denicol Santos, Luísa Todeschini Englert, Márcia Lorena Fagundes Chaves, Brian Lawlor, Bárbara Costa Beber

**Affiliations:** 1 Universidade Federal de Ciências da Saúde de Porto Alegre, Departamento de Fonoaudiologia, Porto Alegre RS, Brazil.; 2 Universidade Federal do Rio Grande do Sul, Faculdade de Medicina, Departamento de Medicina Interna, Porto Alegre RS, Brazil.; 3 Trinity College Dublin, Global Brain Health Institute, Dublin, Ireland.; 4 Universidade Federal de Ciências da Saúde de Porto Alegre, Programa de Graduação em Ciências da Reabilitação, Porto Alegre RS, Brazil.

**Keywords:** Aphasia, Primary Progressive, Speech-Language Pathology, Rehabilitation; Diagnosis, Surveys and Questionnaires, Health Services Accessibility, Afasia Primária Progressiva, Patologia da Fala e Linguagem, Reabilitação; Diagnóstico, Inquéritos e Questionários, Acesso aos Serviços de Saúde

## Abstract

Primary Progressive Aphasia (PPA) is a progressive language disorder associated with frontotemporal impairment and mainly affects the left hemisphere of the brain. In general, this condition compromises abilities related to comprehension and expression of language. The diagnosis of PPA depends on in-depth knowledge regarding functions of language, neurology, and neuropsychology. Speech and language therapists (SLTs) have a pivotal role in the diagnosis and rehabilitation of PPA. The absence of these professionals involved in the diagnosis and rehabilitation may reflect on the quality of care of people with PPA.

**Objective:**

To identify the sociodemographic, educational, and professional practice characteristics of SLTs who work with people with PPA in Brazil.

**Methods:**

An online questionnaire was disseminated to reach SLTs across Brazil. The questionnaire collected information regarding sociodemographics, training and education, practice (time, setting, service provision), and sources of referral.

**Results:**

The study included 71 participants (95.8% women). Specialization was the most frequent educational level followed by master’s degree, and participants where mainly from the Southeast and South regions of Brazil. Neurologists were the professionals who most referred patients with PPA to SLTs. Finally, SLTs worked primarily in homecare settings and provided mainly individual therapy services.

**Conclusion:**

SLTs who work with PPA in Brazil can be characterized mainly as professionals with postgraduate degrees, relatively young, and from the South and Southeast regions of Brazil.

## RESUMO

A afasia progressiva primária (APP) é um distúrbio progressivo da linguagem associado à atrofia de regiões frontotemporais predominantemente do hemisfério esquerdo do cérebro. De modo geral, a APP afeta as capacidades compreensivas e expressivas da linguagem. O diagnóstico depende de profissionais com profundo conhecimento das funções da linguagem, neurologia e neuropsicologia. A fonoaudiologia tem papel essencial no diagnóstico e reabilitação da APP, e a ausência de fonoaudiólogos nesses processos pode refletir na qualidade do cuidado das pessoas com APP.

**Objetivo:**

Identificar as características sociodemográficas, educacionais e de atuação profissional de fonoaudiólogos que atuam com APP no Brasil.

**Métodos:**

Foi distribuído um questionário em formato *online* para fonoaudiólogos de todo o Brasil. O questionário coletou informações sobre aspectos sociodemográficos, de formação, atuação profissional (tempo, local de atuação, tipo de serviço oferecido) e fontes de encaminhamento.

**Resultados:**

O estudo incluiu 71 participantes (95,8% mulheres). O nível educacional mais frequente foi a especialização, e as regiões demográficas com maior incidência de profissionais que atendiam APP foram as Regiões Sudeste e Sul do país. Os neurologistas foram os profissionais que mais encaminhavam pacientes com APP para os fonoaudiólogos. Por fim, os fonoaudiólogos atuavam, principalmente, em *homecare* e realizando, em sua maioria, terapia individual.

**Conclusão:**

Os fonoaudiólogos que atuam com APP no Brasil podem ser caracterizados principalmente como profissionais pós-graduados, relativamente jovens e das Regiões Sul e Sudeste do Brasil.

## INTRODUCTION

Aphasia is a language disorder that results from brain injuries that affect aspects of oral and/or written language associated with semantics, phonology, and syntax, among other language skills^1^. Expressive and/or comprehensive linguistic processes may be altered and affect human communication skills^2^. In general, aphasias can be classified into classic aphasias, crossed aphasias, subcortical aphasias, and primary progressive aphasias (PPA), according to the language symptoms and etiology^2^. The object of interest of this article is the PPA.

PPA is a progressive language disorder associated with atrophy of frontotemporal brain regions involved in language processing^3^. This neurodegenerative disorder, described by Marsel Mesulam in 1982, mainly affects the population between 50 and 65 years old^4^, impairing the comprehensive and expressive capabilities of language in general, which brings significant functional deficits for those individuals^2^. Gorno-Tempini and collaborators described the existence of three PPA variants that manifest themselves through different signs and symptoms, namely: agrammatic (or non-fluent), semantic, and logopenic^3^. In summary, these variants are subdivided according to certain manifestations: the agrammatic variant is marked by apraxia of speech and/or agrammatism; the semantic variant is characterized by fluent spontaneous speech, but with the presence of anomia, deficits in comprehension of isolated words, surface dyslexia or dysgraphia; finally, the logopenic variant is determined by phonological errors, difficulties in finding words and repeating sentences^3^.

PPA diagnosis is carried out through clinical evaluation with the interpretation of several laboratory and neuroimaging tests, in addition to the application of cognitive tests aimed at classifying the disease. PPA manifestations appear during adulthood, initially marked by small impairments in language and later, in a progressive way, by significant functional difficulties in communication, either in expression and/or comprehension of speech. Therefore, it is crucial that the assessment includes tasks that minimally investigate: speech production (grammar and motor speech), confrontation naming, repetition, sentence and single word comprehension, knowledge of objects and people, reading, and writing^3^. The assessment of those linguistic functions must be carried out by a professional with extensive expertise in human language and communication, such as speech and language therapists (SLTs). However, difficulties and delays in diagnosing the disease may occur due to multifactorial factors, and one of them might be the lack of professionals properly prepared to intervene in PPA.

In terms of treatment, there is no pharmacological treatment capable of curing or retarding the course of this language disorder. However, studies are reporting the efficacy and benefits provided by non-pharmacological treatments, such as language rehabilitation^2,5,6^. Therefore, these types of treatments have the purpose of delaying the evolution of the disease and improving the quality of life of this population.

It is clear that SLTs are fundamental professionals in the diagnostic process and responsible for language and speech rehabilitation in cases of PPA^7^, but for this to happen these professionals need proper training and knowledge in language, neurology, and neuropsychology, for example. A study conducted with Brazilian SLTs showed that most SLTs undergraduate courses do not offer neuropsychology courses in their curricula^8^, even though neuropsychology is a specialty area recognized by the Federal Council of SLT in Brazil. As a result, students finish their undergraduate with little or no training in neuropsychology and, consequently, in PPA, which directly reflects the current scenario of lack of knowledge and low investment in the area. In this sense, there are few studies carried out in Brazil on PPA in the field of SLT, with most studies on the subject carried out in the field of medicine, which results in a lack of knowledge about how to plan and execute assessment and language rehabilitation in PPA^9^.

Since SLTs have a central role in the care of progressive language disorders, it is essential to study the profile of SLTs who work with PPA, in order to identify the barriers that make professional performance difficult in these cases and also which are the facilitators for the education of these professionals. In the United Kingdom (UK), investigations have already been conducted to identify patterns and barriers to SLT service provision across the region^10^ and also the practices of those professionals on PPA^11^. However, their results cannot be extended to countries with different socioeconomic and cultural contexts, such as Brazil. It is known that specialized knowledge is critical to fight dementia in inequitable regions like Latin American and Caribbean countries (LACs)^12^, which includes Brazil. Thus, the present study aimed to identify the sociodemographic, educational, and professional practice characteristics of SLTs who work with people with PPA in Brazil in order to contribute to the identification of what needs to be studied and invested in this area in the future.

## METHODS

This study was developed at Universidade Federal de Ciências da Saúde de Porto Alegre (UFCSPA) and it is part of a larger project entitled *“Atuação da fonoaudiologia Brasileira nas demências”*. It was registered and approved by the Human Research Ethics Committee (CEP/UFCSPA), under number 2.733.408. For the development of this research, the requirements of the Brazilian Resolution CNS 466/201211 were met.

Data was collected using an online questionnaire which was distributed to SLTs throughout Brazil and developed in the online tool One Click Survey (www.1KA.si). The questions used for this investigation were based on a questionnaire used in a pilot study conducted in Ireland^13^. The original research contained questions divided into three categories: characteristics of the participants, knowledge about dementia, and awareness of the participation of SLTs in the dementia care process. However, to achieve the objective of the current study, only the following variables were used: gender, age, educational level, demographic region of Brazil in which the professional works, work setting, years of experience as a SLT, years of experience working with PPA, from which professional their patients are referred, and which services they offer as SLTs. Categorical variables were investigated using closed questions while continuous variables using open questions. However, open spaces were offered ways to elicit additional information.

The questionnaire was disseminated on social media (Facebook, Instagram, Twitter) and email lists. With the intention of trying to disseminate the questionnaire equally across all regions of Brazil, the researchers sought collaboration from regional professional organizations and from SLTs with a wide network in each geographic area.

Inclusion criteria were to be a SLT and to be practicing in Brazil at the time of the study. Participants that did not complete the entire questionnaire were excluded from the study.

An online informed consent was presented right at the beginning of the questionnaire presentation, so that participants could read the description of the study and agree or not to participate in the research by clicking on the “I accept” or “I do not accept” button. The questionnaire was only made available to participants who clicked “I accept”. Instructions on how to save or print the informed consent were also provided.

The results of this study were presented in a descriptive way. Continuous variables were described as mean and standard deviation, while categorical variables were described as absolute frequency (n) and relative frequency (%).

## RESULTS

**Figure 1.**
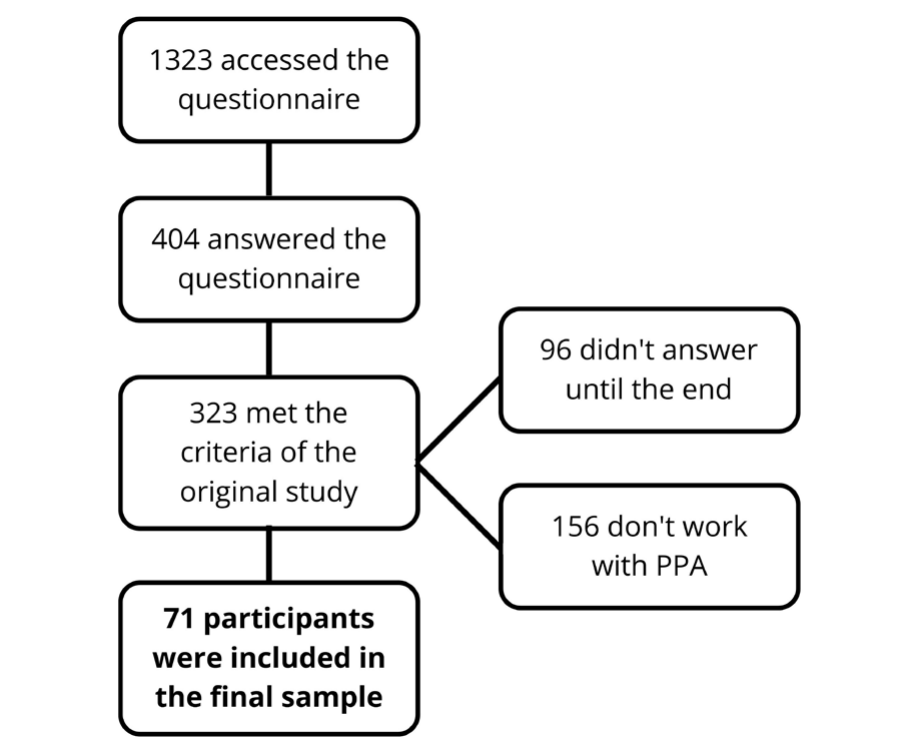
Flowchart for sample selection.

The present survey had 1,323 visitors, but after applying the inclusion and exclusion criteria, 71 were included in the sample (Figure 1). The sample consisted mostly of young women with about one decade since the completion of their undergraduate course, but with fewer years of experience with PPA. Most participants had some type of postgraduate degree (Table 1).

**Table 1.** Description of the sociodemographic characteristics of the sample.

Characteristics		N (%) or Mean (SD)
Gender (F) – N (%)		68 (95.8)
Age (years) – Mean (SD)		38.12 (9.98)
Training time (years) – Mean (SD)		11.58 (10.86)
Time of experience with PPA (years) – Mean (SD)		6.45 (7.42)
Academic degree – N (%)	Specialization	33 (46.5)
	Master’s degree	12 (16.9)
	Undergraduate	10 (14.1)
	PhD	9 (12.7)
	Residence	5 (7)
	Post-doctoral	2 (2.8)

Abbreviations: SD, standard deviation; PPA, primary progressive aphasia.

**Figure 2.**
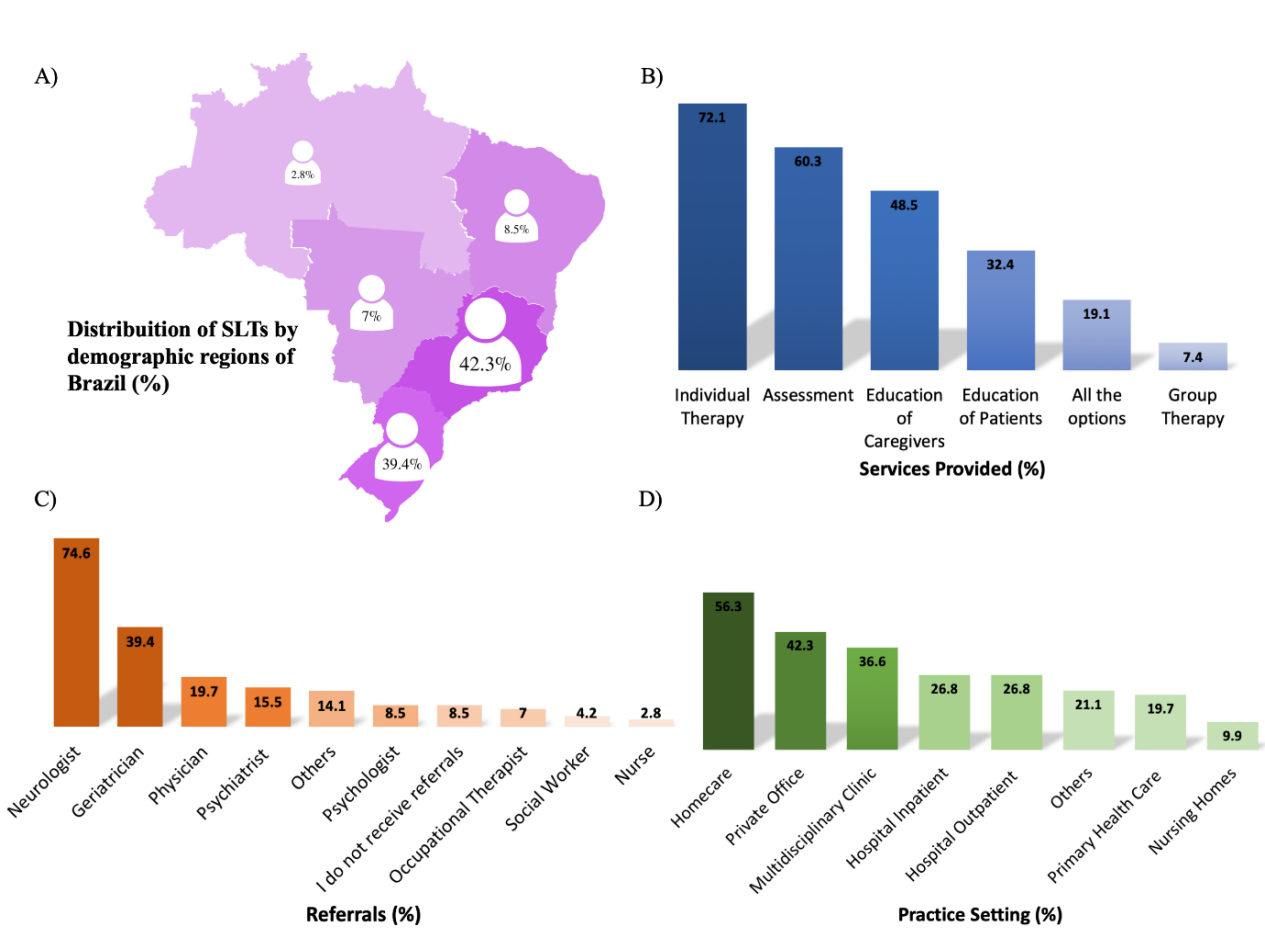
Distribution of speech and language therapist by geographic region (A), services provided (B), sources of referral (C), and practice setting (D).

Abbreviation: SLTs, speech and language therapist.

The demographic regions with the highest numbers of professionals who deal with PPA were the Southeast and South, respectively (Figure 2A). Individual therapy was the most commonly provided service by SLTs for people with PPA, following the assessment of speech and language skills. On the other hand, group therapy appeared to be the least provided service by those professionals (Figure 2B). Regarding the professionals who refer patients with PPA to SLTs, neurologists were the most cited professionals, followed by geriatricians (Figure 2C). The most frequent practice setting of SLTs that work with people with PPA was at home and in a private office practice, with a lower frequency in nursing homes (Figure 2D).

## DISCUSSION

This study was aimed at identifying the sociodemographic, educational, and professional practice characteristics of SLTs who work with PPA patients in Brazil. Most participants had postgraduate degrees and were mainly from the Southeast and South regions of Brazil. Neurologists were the professionals who most referred patients with PPA to SLTs. SLTs worked primarily in homecare settings and provided mainly individual therapy services. We believe that these findings may help to recognize the obstacles and facilitators of the performance and preparation of those professionals in the care of people with PPA, and to point out the direction of future investigations.

The most prevalent educational level of Brazilian SLTs who work with PPA was mainly specialization and master’s degree, demonstrating that, possibly, these professionals seek additional training after undergraduate to feel qualified to work. It is possible that this is due to the absence of training in the area of neuropsychology during undergraduate in SLT^8^, added to the lack of discussions and research about PPA in the academic environment^9^. Thus, the assessment, diagnosis and treatment of PPA depend on a trained professional with regard to knowledge of neuropsycholinguistic processes^9^. This is still an underexplored area in the Brazilian SLT undergraduate courses, which implies the need for specializations and studies that go beyond undergraduate courses.

Neurologists were the professionals who referred the most patients with PPA. In the study conducted in the UK, neurologists were also the main source of referrals, but with a much lower percentage (22.5%)^10^ when compared to the percentage in Brazil (74.6%). It is possible that in Brazil there is a lack of awareness about the role of SLTs in the care of PPA people among other health professionals.

In addition, the present study found that the most prevalent SLT practice setting was the patients’ own homes, or as it is known, the homecare setting. This result probably reflects the structure of Brazilian SLT care in general. However, it may also be related to the late diagnosis of PPA in Brazil. When diagnosis is delayed, patients may be very compromised by the disease and unable to carry out simple activities^9^, such as leaving home to go to therapy — which makes home care a strategy that expands the access to treatment for patients in advanced stages of dementia. However, despite being a facilitator for treatment, the high rate of SLTs who treat PPA patients in this setting reflects a situation of general lack of knowledge in the area, as it is likely to be a result of late diagnosis. Thus, it is evident that currently the PPA diagnosis suffers from a series of difficulties and delays to be completed^9^, which leads to a worsening of the neurodegenerative condition of the disease, thus making therapeutic intervention difficult^14^, since the main speech therapy intervention in PPA consists mainly of delaying the progression of communication deficits. Another point that must be taken into account is the small number of professionals who use group therapy as a rehabilitation strategy. In this sense, although some studies prove the effectiveness of this type of therapy, especially with regard to the quality of life and depression of patients with dementia^15,16^, there are still few investigations in this area, even less in the Brazilian population and with people with PPA. It is known that frontotemporal dementia is a low-frequency dementia, which is likely under- or late diagnosed^17^. Those factors may justify the difficulty to offer group therapy as a rehabilitation strategy for patients with PPA.

Finally, the investigation demonstrates a greater concentration of Brazilian SLTs who work with PPA in the South and Southeast regions of the country, while in the North, Central West and Northeast regions the rates are quite low. This result may be related to the low number of SLTs, in general, in these regions^18^. On the other hand, it may reflect the lack of investment and opportunities for SLTs in those regions when compared to the South and Southeast, which has already been described in the literature^19^. It suggests an inequitable access to specialized care for people with PPA in Brazil, which is not seen in developed countries such as the UK, where the distribution of SLTs who work with PPA is much more balanced^10^.

The study’s limitation is the fact that recruitment was carried out by researchers from southern Brazil, which may have facilitated the recruitment of participants from this region, even with all the efforts to make recruitment equitable. On the other hand, it is known that the number of SLTs working in the South and Southeast is much higher than in the rest of the Brazilian territory, which may also explain the divergence between the number of professionals surveyed who work in different regions.

In conclusion, SLTs who work with PPA in Brazil can be characterized mainly as professionals with postgraduate degrees, relatively young, and from the South and Southeast regions of Brazil. Regarding their clinical practice, the professional who most often refers people with PPA to SLTs is the neurologist, and most SLTs perform individual therapy in a homecare setting. Improvements in access to training in this area, more homogeneously and systematically, could raise the quality of professional practice in the future. In depth, there is a need to improve the training of SLTs, especially during undergraduate courses, on PPA, which could result in more professionals prepared to intervene in this field in different geographic areas of Brazil, offering a greater diversity of services in more diverse work settings. This should also facilitate the inclusion of SLTs in memory clinics supporting earlier diagnosis and intervention on PPA, since this is within the scope of SLT. Further studies and research are still necessary, since scientific production on the subject is still scarce in Brazil.
